# HOME protocol for a national online survey of people who inject drugs

**DOI:** 10.1186/s12954-025-01260-6

**Published:** 2025-07-18

**Authors:** Winston E. Luhur, Cristina L. Chin, Jazmine M. Li, Grey Marsh, Dan Coello, Aaron Fox, Honoria Guarino, Denis Nash, Viraj V. Patel, Czarina N. Behrends

**Affiliations:** 1 Department of Population Health Sciences, Weill Cornell Medicine, New York, NY USA; 2 NEXT Distro, New York, NY USA; 3 Division of General Internal Medicine, Department of Medicine, Albert Einstein College of Medicine, Montefiore Health System, Bronx, NY USA; 4 Institute for Implementation Science in Population Health, City University of New York (CUNY), New York, NY USA; 5 Department of Epidemiology and Biostatistics, CUNY Graduate School of Public Health and Health Policy, New York, NY USA

## Abstract

Most surveys of people who inject drugs (PWID) fail to represent the full population of PWID, because usual recruitment methods do not achieve geographic and sociodemographic diversity. People of color, people residing in rural and/or harm reduction-deprived areas, and people who rarely connect with social services are the least surveyed and understood PWID populations. Online-based recruitment and surveys may better reach these hidden PWID populations than standard venue-based recruitment. As technology use and internet access become more ubiquitous, even for unstably housed populations, research using online-based recruitment and survey techniques are growing in the substance use field. These methods hold promise for obtaining larger and more diverse PWID samples, but there are no standards for using online recruitment and survey administration methods to reach large populations of PWID vulnerable to overdose and other threats. Best practices are needed to maximize data quality, prevent fraudulent responses, and minimize selection biases. The HOME (Harm reduction services Offered through Mail-delivery Expansion) study recruits and enrolls a national, online-recruited, longitudinal cohort of 1233 PWID and follows them for 18 months. Key objectives are to assess prior harm reduction utilization and future uptake of mail-based harm reduction services and retention in these services. We describe our online data collection protocol, including recruitment approaches, detecting fraud, maximizing data quality, and participant retention throughout follow-up. These strategies can inform subsequent large-scale, nationwide efforts that recruit PWID through the internet.

## Introduction

Rapidly increasing use of fentanyl and other synthetic opioids [1] and now xylazine (“tranq”), has exacerbated drug-related morbidity and mortality in the United States (US) [2, 3]. Opioid use disorder (OUD) is one of the largest public health crises affecting about 5.7 million people in the US aged 12 years or older (2.0% of population) in 2023 [4]; in addition to age-adjusted fentanyl-related overdose death rates more than tripling between 2016 (5.7 deaths per 100,000) and 2021 (21.6 deaths per 100,000) [5]. The state of opioid use and overdoses in the US has created urgency to increase access to harm reduction services for people who inject drugs (PWID), including distribution of new syringes, fentanyl test strips, and naloxone, an over-the-counter drug that can reverse opioid overdoses.

In the US, the main options for obtaining sterile syringes and naloxone are pharmacies and syringe services programs (SSPs), but these venues have limitations. PWID may avoid using pharmacies where they experience stigma, staff may lack education about dispensing policies, and/or the pharmacy fails to stock supplies [6–12]. Inconvenient hours or distant locations, unwarranted interference by law enforcement, lack of transportation, and/or fear of stigma may also prevent PWID from using in-person syringe services programs (SSPs) [13–15]. Half of PWID are estimated to live outside of major urban areas [16], yet 69% of SSPs are located in urban settings [17]. Among all PWID in the US, only 36% have syringe coverage via SSPs [18]. Many PWID cannot entirely rely on the current options for all their harm reduction needs.

Mail-based harm reduction services may expand harm reduction access for underserved PWID. Needle Exchange Technology (NEXT) Distro is a national, online-based, non-profit harm reduction program that mails out naloxone, syringes, and other supplies typically found at SSPs [19–21]. Online-based, mail-delivered services can mitigate transportation barriers and improve access in poorly resourced rural areas while improving privacy and confidentiality; however, with disparities in internet access in the US, questions remain about whether online-based mail-delivered harm reduction services can equitability reach underserved populations of PWID. It is also unclear what factors make PWID more likely to initiate and continue to use online-based mail-delivered harm reduction services over time thereby decreasing substance use-related risks.

Online-based research with PWID also creates the opportunity to improve survey methods for reaching underserved PWID. Venue-based recruitment, where PWID are enrolled at SSPs or clubs and festivals, results in a sample with limited demographic and geographic diversity, particularly with regards to rurality, race, ethnicity, and gender [22–25]. The National Survey on Drug Use and Health (NSDUH), a nationally representative household survey for substance use in the US [26], only targets non-institutionalized civilians and likely underrepresents PWID experiencing housing instability or living inside institutions (e.g. hospitals and jails). Other national surveys that target PWID, such as the National HIV Behavioral Surveillance (NHBS) [27], are limited by recruitment methods (i.e., respondent-driven sampling) that limit sample diversity and geographic reach. Finally, these surveys do not assess harm reduction access and utilization in detail. As the use of the internet and mobile devices have grown more ubiquitous, online-based recruitment has been successful in recruiting national cohorts of hidden populations, such as transgender people [28, 29] and those at high risk for HIV [30–32]. However, despite the promise for recruiting diverse national samples, there has been limited application of these methods to reach PWID.

The HOME (Harm reduction services Offered through Mail-delivery Expansion) study aims to inform scale-up of mail-based harm reduction services in the US. This manuscript describes the protocol for Aim 2 of the HOME study: a national, online-based longitudinal cohort of adult PWID in the US who have not used mail-based harm reduction services in the past 6 months to assess predictors of uptake and retention into mail-based harm reduction services. Other HOME study components—examining policy barriers to mail-based harm reduction services and service preferences for mail-delivery clients—are not presented here. With this survey, we aim to produce new methodological knowledge regarding online-based recruitment and survey administration with PWID, which may contribute to best practices of these emerging methods. We describe our online-based recruitment and study protocol here to provide an approach for developing online-based cohorts with PWID. We provide details on our recruitment approach, tactics to minimize fraudulent responses and sampling biases, and considerations for achieving a diverse PWID sample. 

## Methods

### Study overview

#### Background

This study aims to enroll, using online recruitment methods, a convenience sample of 1233 adult PWID located in the US who have not used mail-based harm reduction services in the past 6 months. We have targeted sampling goals to obtain a sample that has sufficient representation of Black PWID, Hispanic PWID, women who inject drugs, and rural PWID to allow for subgroup analysis of these traditionally underserved PWID populations. Participants will complete screening, consent, and baseline surveys online, then they will be sent information about NEXT Distro with a unique link to sign up for mail-based harm reduction services as well as information on locations of syringe services programs. After the baseline survey, participants then complete online-based surveys at 3, 6, 12, and 18 months. We then examine uptake of mail-based harm reduction services, characteristics associated with retention, and changes in substance use behaviors. This research was approved by Weill Cornell Medicine IRB.

#### Community advisory board and stakeholder engagement

This project is designed and conducted in close collaboration with NEXT Distro and a community advisory board (CAB) that is established for the study. The CAB includes five to ten NEXT Distro program affiliates and clients with lived experience of drug use. The CAB convenes with the study team at least once per year providing input and advice on various aspects of the study, such as study advertisement imagery and messaging, online locations to advertise the survey for recruitment, and survey content, terminology, and length.

### Inclusion criteria

Participants are eligible to be part of the study if they are located in the US, 18 years or older, English- or Spanish-speaking, have injected drugs (not as prescribed) in the past 30 days, and have not used mail-based harm reduction services in the past 6 months. Participants also receive questions that test for injection drug use knowledge and are screened against fraud detection protocols—detailed below—to confirm eligibility.

### Recruitment

#### Advertisements

Participants click on a study advertisement which links to the eligibility survey. In collaboration with our full study team, NEXT Distro colleagues, and the CAB, we developed the study name (HOME), logo, a study website, and ideal messaging and designs for the advertisements. Different versions of study advertisements use graphics from NIDA’s publicly available photo album and NEXT Distro, which showcase harm reduction supplies such as syringes, naloxone, and other safe drug use supplies. Advertisements contain slight variations in language and graphics that are suited for different kinds of social media platforms and formats. The study advertisements, available in English and Spanish, emphasize confidentiality, incentive amounts, and the overall goal of the study.

#### Online recruitment settings

We promote the study across relevant websites and social media platforms that may engage PWID. Settings include websites that offer drug use content and forums for discussing drug use (e.g., Bluelight, Drugs-forum), subreddits and online discussion groups focused on drug use (with permission from the moderators), and large social media venues (e.g., Reddit, Instagram, TikTok, etc.). We plan to reach out to national drug user unions to advertise through those organizations. Harm reduction organizations and SSPs are not targeted for recruitment as this may select for a sample that already has access to harm reduction services and we seek a more diverse set of experiences with harm reduction in this sample. However, we plan to advertise to PWID who are currently on the waiting list of NEXT Distro as these individuals have not yet initiated mail-based harm reduction services with NEXT Distro.

To improve the reach of our study advertisements to underrepresented populations of PWID, we advertise on platforms that have higher utilization by minority populations, such as TikTok and YouTube, which are popular among young Latinx and Black groups [33]. Moreover, we have Spanish language advertisements that allow us to advertise the study on Spanish-language based platforms oriented towards drug use. Different platforms also allow for customization that target advertisements to specific populations, which we employ when needed to reach our recruitment targets. We regularly monitor race/ethnicity, gender, and rurality during recruitment to determine if there is under sampling by group so that we can adjust our advertising to be more focused on recruiting these subgroups.

To increase our legitimacy and engender trust among prospective participants, we create study social media accounts on major platforms such as Facebook, Instagram, Twitter/X, Discord, and Reddit. Study staff regularly post promoting our study and harm reduction in general by re-sharing harm reduction resources such as those from North America Syringe Network (NASEN) and NEXT Distro. Potential participants may learn about the study through our posts and get linked to the eligibility survey by clicking on those posts or visiting our website. Potential participants can also learn more about our study through the study website, which provides the study description, profiles of the study investigators, and links to more resources alongside a link to the eligibility survey.

#### Recruitment strategy

**Fig. 1:**
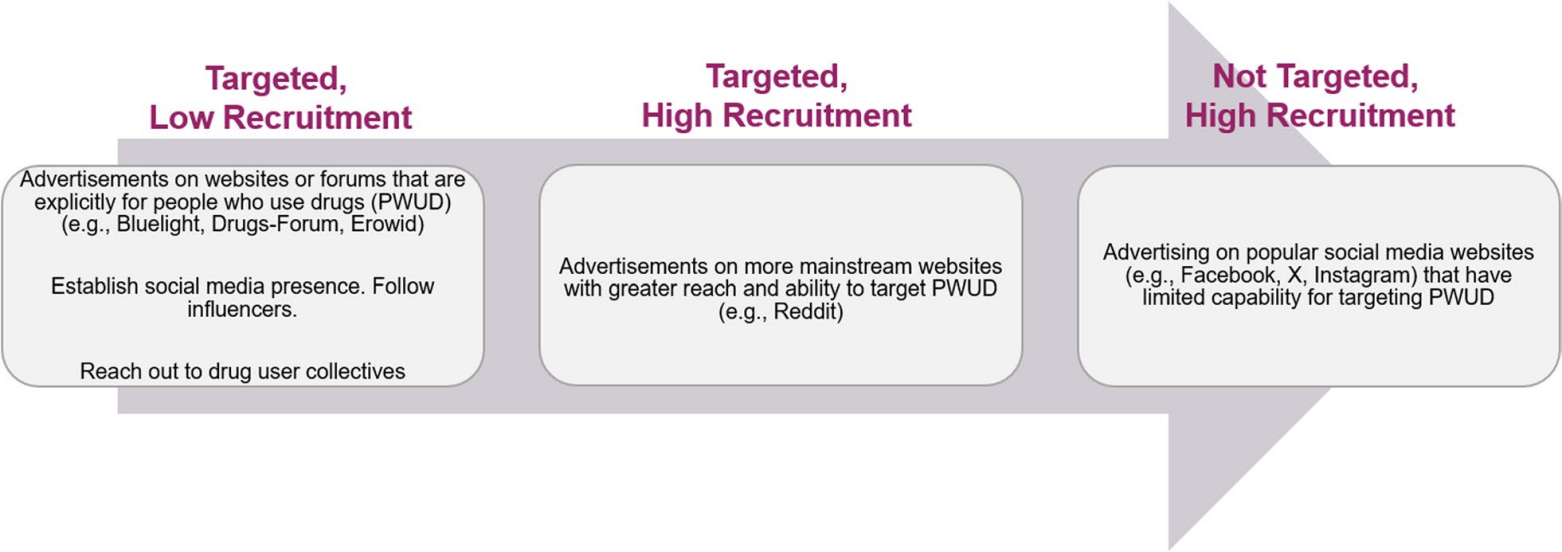
HOME three-stage recruitment strategy

Marketing occurs in three stages: (1) targeted, low traffic recruitment strategy, (2) targeted, high traffic recruitment strategy and (3) non-targeted, high traffic recruitment strategy (Fig. 1).

The first 6 months following study launch use the targeted, low traffic recruitment strategy by posting advertisements to websites designed for people who use drugs, such as Bluelight.org. For Bluelight, advertising occurs via a site-wide banner advertisement, forum posting, and an announcement on Bluelight’s Discord server directing members to take the eligibility survey. These websites are likely to reach PWID, but we anticipate lower recruitment numbers than other strategies due to the lower traffic of these websites in comparison to larger social media platforms. This first strategy employs a slow ramp up of recruitment allowing for testing of advertisements, survey links, and fraud detection protocol. In this stage, each eligibility and study survey can be examined on an individual basis to verify that the fraud detection tools are working.

The second stage builds on the first stage and includes a targeted and high traffic recruitment approach that advertises on a mainstream platform where we can target advertising to people who use drugs. This stage focuses on advertising on Reddit and targeting relevant subreddits such as r/drugs, r/opiates, r/Fentanyl, r/HarmReduction and any other similar Reddit communities that have a sizable following and activity of PWID. Moderators of these groups are contacted by the study team to allow posting of study information on the subreddit. We prioritize subreddits that have a previous history of allowing for study advertising.

The last strategy builds on the first two strategies and includes a non-targeted, high traffic recruitment approach that advertises on popular social media websites (e.g., Facebook, Instagram, X/Twitter, TikTok, Tumblr). These websites do not necessarily allow targeted advertising toward people who use drugs, but the large traffic should increase visibility of advertisements. Some of these platforms do include algorithmic advertisement targeting based on people who click on the advertisements, but this algorithm still may not necessarily target people who use drugs. This is the last stage in our recruitment approach as we anticipate these major platforms pose a higher risk of bots or fraudulent entries. Depending on engagement levels, we may also identify social media influencers in the harm reduction or drug use online communities, especially Black, Latinx or women influencers, and pay them to promote our study within their network.

#### Measuring the yield of recruitment strategies

**Fig. 2:**
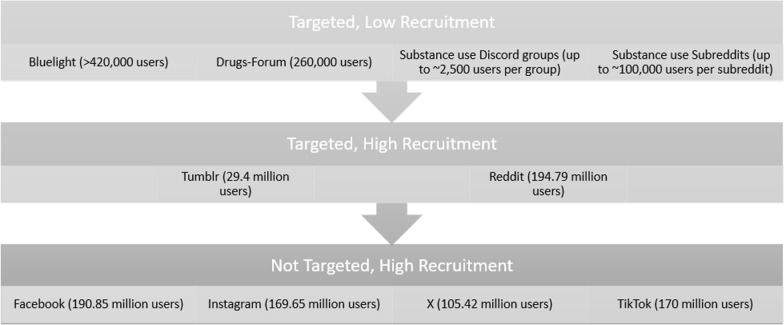
Recruitment venues and potential reach

The potential reach of each online advertising venue is presented in Fig. 2. We track advertisement engagement metrics by creating unique links for each advertisement campaign that details the visual design, content, and location where it was advertised. We use tracking metrics through Bit.ly and Qualtrics to determine which version results in the most clicks, completed eligibility surveys, and highest percentage of eligible participants. We also monitor these metrics to determine which recruitment strategy is most successful. Analysis of these metrics include examining the specific visual design, written content, and advertising platform and its association with (1) number of eligibility surveys completed and (2) percentage of eligibility surveys that result in eligible participants. Diversity yield by recruitment strategies is assessed by looking at sociodemographic characteristics of eligible participants. Yield data collected across recruitment stages and at the end of baseline recruitment informs the advertising approach throughout the study and identifies the most effective strategies for online recruitment of PWID.

### Eligibility survey

By clicking on the advertisements or on the link provided on the study website, prospective participants are linked to the web-based eligibility survey that collects self-reported sociodemographic information (race, ethnicity, age, gender identity, education, urbanicity, state of residence), drug use history, and contact information (email, phone number, social media handles, alternate contacts). Participants can complete the eligibility survey in English or Spanish. Participants must also answer questions designed to test familiarity with drug use terminology (see *Fraud detection*). Provided no fraud detection flags are triggered (see *Fraud detection*), and the participant meets eligibility criteria, they are sent an email with a personalized link to the first part of the baseline survey within 2 business days.

### Informed consent

Informed consent is provided before the start of the survey by presenting eligible participants with study information and potential risks and benefits. Afterwards, participants must score 100% on two multiple-choice and two true/false reading comprehension questions based on the consent document they just read. The purpose of including reading comprehension questions in the informed consent process is two-fold: (1) to ensure the participant understands the informed consent document and (2) determine if the participant requires staff assistance to take the survey due to low reading comprehension [34, 35]. Participants are given two opportunities to pass the informed consent comprehension questions; those who do not pass all four questions are contacted by a staff member to assist them with taking the survey in their preferred language (English or Spanish) over the phone or by video conference.

### Survey workflow

To reduce survey fatigue, especially considering the unique needs among PWID [34], we split the baseline Qualtrics survey into two parts to keep the surveys short, each taking 10–20 min to complete. The second part of the baseline survey is sent after the first part is completed. Once the second part of the baseline is complete, participants are asked if they want to continue to the NEXT Distro intake form to potentially order harm reduction supplies. Otherwise, participants exit the survey. Participants receive a thank you email with a link to the incentive (if eligible) after completion of each survey. They also receive links to NEXT Distro and a national list of SSPs by geography. Participants who do not complete both baseline surveys after four follow-up attempts for each survey are excluded from the cohort study and considered lost-to-follow-up. Follow-up surveys are sent at 3-, 6-, 12-, and 18-months post-baseline.

### Survey development

The survey design is informed by a conceptual framework by Levesque et al. [35] on access to healthcare to frame potential predictors and/or confounders of service access and engagement. The surveys include questions to assess participants’ (1) *ability to perceive* (i.e., trust, digital literacy), (2) *ability to seek* (i.e., culture, gender), and (3) *ability to reach* (i.e., reliable source of internet and technology access and a place to mail supplies to), but primarily focus on (4) *appropriateness/ability to engage*, which includes measures of adherence, transportation, and housing (Table 1).

**Table 1:** HOME survey measures and outcomes

Category	Measures	Levesque Access to Healthcare Domain
Sociodemographic characteristics	Age, gender identity, race/ethnicity, sexual orientation, income source, housing status, employment, health insurance	Ability to Seek and Reach
Geographical area	Rural vs. non-urban categorization by ZIP code	Ability to Seek and Reach
Substance use	Ever used, age of first use, use in past month	Availability and Accommodation/Ability to Reach
Injection drug use	Age of first injection drug use	Availability and Accommodation/Ability to Reach
Assessing behaviors associated with injection drug use	Equipment reuse and sharing in past month, skin and soft tissue infection occurrence in past month	N/A
Access to equipment	Amount and where one gets new/unused syringes or pipes in past month	Availability and Accommodation/Ability to Reach
Access to and utilization of services (syringe services programs [SSP]/needle exchange, overdose prevention centers)	Utilization of harm reduction services, convenience of accessing harm reduction services (distance)	Availability, Accommodation, and Appropriateness/Ability to Reach
Acceptability and willingness to use each of the following services: 1) mail delivery, 2) SSP, 3) pharmacy	Likert scale of acceptability and willingness to use for each service	Acceptability/Ability to Seek
Access to harm reduction equipment	Utilization of drug checking, amount and where one gets naloxone/Narcan kits	Availability and Accommodation/Ability to Reach
Social media/internet access	Internet access (capability, frequency, connection stability), devices used for Internet access, social media utilization	Availability and Accommodation/Ability to Reach
Overdose experiences	Overdose experience frequency and symptoms	N/A
Self-directed actions to reduce risks associated with drug use	Utilization of naloxone/Narcan, frequency of drug use where one is in presence of naloxone/Narcan, have taken steps to minimize risk of overdosing	Ability to Engage
Substance use disorder treatment	Ever received in past 6 months or currently receiving and what kind	N/A
HCV	Status, received treatment	N/A
HIV	Status, ever taken PrEP, currently taking ARV	N/A
Criminal legal system involvement	Direct contact with police, arrests, held in jail or prison, been on probation or parole	N/A
Likelihood of arrest for utilizing harm reduction services or having new/unused syringes	Likert scale of perceived likelihood of arrest	Ability to Seek and Reach
Experienced and perceived stigma	Substance Use Stigma Mechanisms Scale (SU-SMS)	Acceptability/Ability to Seek
Initiation (uptake) of mail-delivered harm reduction services	% of sample with first time use of NEXT	Appropriateness/Ability to Engage
Harm reduction retention (3, 6, 12, 18 months)	Utilization in the past 6 months of follow up (12 and 18 month) among people who had initiated NEXT use	Appropriateness/Ability to Engage

The survey questions are primarily developed with the Harm Reduction Research Network [36], a national network of research projects funded by the National Institutes of Health to study the effectiveness, implementation, and impact of harm reduction policies and interventions. The coordinating site of the Harm Reduction Research Network facilitated the harmonization of common questions across all ten funded harm reduction projects, including the HOME study. Meetings were held regularly to review proposed questions and achieve consensus on questions to harmonize, including the wording of these questions. Our survey includes the primary harmonized questions and additional secondary questions that are important to answer our research questions. Our survey incorporates input from our research team and our CAB members on the wording of questions and survey length.

### Survey incentive

The participant’s baseline survey incentive is sent after each part of the baseline survey is completed, and no fraudulent responses are identified nor issues necessitating follow-ups are needed. In total, participants receive $30 for completing both parts of the baseline survey with $15 provided upon completion of each baseline survey. The same process is repeated for completion of each one of the follow-up surveys; the 3-month and 6-month surveys garner a $20 incentive each, while the 12- and 18-month surveys provide a $40 incentive to aid in study retention. All participant incentives are provided via a unique redemption link to tangocard.com where they can select their digital incentive format (e.g., Visa, vendor-specific digital gift card, or direct deposit into Venmo). The study team verifies that all survey submissions are not bots or repeat survey participants (i.e., taking the same survey multiple times to get multiple incentives) to assess incentive eligibility.

### Retention plan

Given the extended duration of the study (18 months), we are implementing a series of strategies aimed at maximizing participant retention and survey completion rates. The first strategy involves a tiered incentive structure designed to encourage ongoing participation (see *Survey incentive*). Secondly, eligible participants are sent weekly reminders via email or text message to take the survey, including those who have been deemed eligible but have not yet completed the baseline survey, those who initiated but did not complete a survey, and those approaching their next follow-up survey. If provided, we will also message the participant’s social media account and/or the contact information of a person in their network. Reminders at each stage are sent up to three times; afterwards unresponsive participants are deemed lost-to-follow-up.

## Fraud detection

### Eligibility survey

Due to the online, anonymous nature of the survey, coupled with monetary incentives for completing the survey, fraudulent responses are a major concern [37–39]. The fraud detection strategies aim to (1) block non-legitimate VPN and proxy server use [40], (2) block non-US participants, (3) prevent multiple submissions from the same participant, (4) exclude bots [41], and (5) ensure actual PWID are taking the survey [42].

First, we implement an IP address block for participants under a VPN or proxy server and anyone whose IP address originated from outside the US by integrating tools provided by IPHub.info into the Qualtrics surveys [43]. At the beginning of the survey, participants are informed that they will not be able to complete the survey using a VPN or proxy and must disable these features. Positive matches for using a VPN or proxy or not residing in the US are automatically kicked out of the survey. Participants using a VPN or proxy receive a prompt that instructs them to return to the survey with their VPN or proxy turned off. These procedures were tested with commercially available VPNs (Mullvad, Nord VPN, and Private Internet Access).

Multiple survey submissions by the same participant are detected using the Qualtrics built-in Ballot Box Stuffing and RelevantID scores [44]. Participants assigned the value TRUE from the Ballot Box Stuffing tool and/or 100 for the RelevantID score indicate that they may have completed the survey previously, even if the IP addresses are different. These participants are allowed to continue the survey but are flagged in the dataset to be checked manually by research staff. We do not use these scores to automatically deem a participant ineligible to allow for participation from multiple individuals who may be sharing one device. To help in our determination of fraudulent activity, a prompt in the eligibility and study surveys ask if the participant is taking the survey from a personal device solely operated by themselves, a shared device, or a mix of both.

Suspected bots are screened out by using the Qualtrics built-in reCAPTCHA prompt at the beginning of the survey. CAPTCHA is a non-text-based bot detection that alleviates bots [45, 46]. Those who failed the prompt are automatically kicked out of the survey with their responses marked as unfinished.

Lastly, to ensure the enrollment of actual PWID, the eligibility survey includes domain knowledge questions meant to assess knowledge of injection drug use [47, 48]. For questions on drug use history, a honeypot response option “Panax” is included as a non-legitimate response. Questions on equipment used to prepare drugs include the non-legitimate response “Tape.” A free text entry for preferred needle size for injecting drugs is used as a check for knowledge of injecting drugs. This method is our best attempt to ensure that our participants are PWID; due to the nationwide sample, we are not able to tailor these questions to local drug packaging or knowledge questions that have been used in prior research [42]. Additional verification of participants’ device information, emails (e.g., sequential, or nonsensical addresses), phone numbers (e.g., VoIP numbers), social media handles, as well as examining very short survey completion time (outliers) are performed prior to enrolling eligible participants into the baseline survey.

#### Post-hoc

As noted in detail above, we check responses to the eligibility survey questions to ensure data validity (e.g., VPN/proxy/non-US detection, multiple submissions, bots). After the completion of the baseline surveys, we also do consistency checks of sociodemographic characteristics between the eligibility survey and the baseline survey to ensure internal consistency. If there is inconsistency detected, we reach out to the participant directly to resolve it. We also monitor free-text responses and responses to scale-based questions to detect suspected fraudulent responses that escaped all of our existing fraud detection protocols. Additional post-hoc checks are planned once we have enough data to analyze. We plan to implement machine learning algorithms to detect human or sophisticated bots who can produce legitimate-sounding responses [41, 49].

#### Outcome definitions

The analytic goal of this study is to examine predictors of uptake and long-term engagement in mail-based harm reduction services among a national, online-recruited cohort of PWID. Uptake is defined as any use of NEXT Distro in the 18 month follow up with the whole cohort as the denominator. Long term engagement is defined as either 12-month retention or 18-month retention. Participants who initiate use of NEXT after the baseline survey and utilize NEXT at least once within 12 months of their first NEXT program use are considered as having 12-month retention. Participants with 12-month retention who use NEXT at least once between 12 and 18 months of their first NEXT program use are considered as having 18-month retention.

#### Analytic plan

Data from the surveys are reviewed and summarized using descriptive summaries (e.g., percentages, means with standard deviations, median, and range) and graphical analyses to ensure that values are within appropriate ranges, to check for outliers and abnormal values, and to verify that the distributions of measures meet the assumptions of the statistical tests that are planned. Linear, Poisson, or logistic regression will be employed to assess hypotheses as appropriate and include potential confounders. We have two main hypotheses to test below.

*1) Hypothesis on mail-based harm reduction uptake at 12 and 18 months:*

We hypothesize that women and rural PWID are significantly more likely than others to initiate use of mail-based harm reduction services within 12 or 18 months. This hypothesis is supported by high representation of women in NEXT Distro services compared to SSPs (53% vs. 39%, respectively) [50, 51] and only 28% of NEXT participants who inject having previously received syringes from safe sources [52].

The association of women (vs. men) with the outcomes of uptake (any use of NEXT in the 12 month follow up vs. not; any use of NEXT in the 18 month follow up vs. not) are assessed using logistic regression. A second logistic regression model measures the association between rurality (vs. urbanicity— using Rural–Urban Commuting Codes to identify rural/urban designation) and the same outcomes above. Potential confounders include race/ethnicity, age (under 30 years vs. 30 years or older), drug of choice, stigma, housing status, local harm reduction availability, risk behavior, Internet access, and digital literacy. Assessment of confounding or effect modification a priori to inclusion in the model is assessed by the study team with framing from the Levesque et al. conceptual model. Each potential confounder and its univariable association with the outcomes are estimated, and potential confounders with p-values greater than 0.10 are not included in the multivariable model unless there is a priori rationale for inclusion. Non-significant variables with small outcome effects (< 10% change) are removed from the final multivariable model to improve model efficiency. Results include reports of beta coefficients and odds ratios.

*2) Hypothesis on long-term retention: PWID with no regular harm reduction source (SSP/pharmacy) are more likely to be retained in mail-based harm reduction services at 12 and 18 months*:

Using logistic regression, we examine the association of harm reduction access (regular access/utilization of any harm reduction program vs. no regular harm reduction source) with the outcome of retention (12-month retention vs. not; 18-month retention vs. not). Potential confounders include: race/ethnicity, gender, geographical area (rural, suburban, urban—self-identified and verified by ZIP code), age (under 30 years vs. 30 years or older), drug of choice, stigma, housing status, transportation, local harm reduction availability, risk behavior, Internet access, and digital literacy. Analysis is conducted using the same methods reported for the first hypothesis above.

Secondary analyses includes three different analyses of how use of mail-based harm reduction services is associated with the following outcomes (with analytic approach): (1) syringe coverage defined as the percentage of injections that uses a new syringe (Poisson regression), (2) risk behavior defined by syringe sharing in the past 3 months (logistic regression), and (3) the utilization of harm reduction services defined as the average utilization per month (linear regression). Subgroups, such as people with no regular harm reduction sources, are examined in exploratory analysis. These primary and secondary outcomes elucidate whether there are differential levels of risk and program engagement among specific underserved populations. This will inform future targeted efforts to improve engagement, which is important if these programs are to be expanded to meet the needs of underserved populations.

#### Missing data

Attrition is assessed by risk behavior and sociodemographic factors. For missingness not at random, we will employ sequential regression multiple imputation to impute missing values [53]. This method uses a combination of multiple imputation and inverse probability weighting (IPW), with IPW used to address the exclusion of individuals with a lot of missing variables as a result of non-participation that may occur in longitudinal studies [54]. This would be to address some limitations of multiple imputation that relies on joint probabilities, which if mis-specified with many missing variables could cause bias.

#### Sample size

Online-based recruitment reaches a large number of people quickly (e.g., > 1000 participants within a month in HIV prevention studies) [55], facilitating oversampling for Black, Latinx, women, and rural PWID populations that are usually hard to recruit in community or venue-based studies. Previous internet-based surveys have recruited upwards of 4000 of Black/Latinx individuals out of a larger sample of nearly 9000 in HIV studies using the same approach as described in this study [56, 57]. For our primary analysis, we will aim to recruit a sample of 1233, assuming 15% loss of the baseline enrollment to fraudulent entries, resulting in a baseline recruitment of 1048 participants [58, 59]. We then assume that 70% of sample remains in the study at 18 months based on attrition in previous studies with PWID [60–62]. This would result in a retained cohort of 734 participants. People who miss a follow up may participate in the study at a later follow-up time to account for institutionalization (i.e., incarceration). Differential lost-to-follow up is assessed to determine potential bias and generalizability.

This sample powers univariate and multivariate logistic regressions that examine predictors of long-term (12 and 18 month) retention of mail-based harm reduction services with sufficient power (~ 80%) to detect a moderate odds ratio of 1.8 (95% confidence interval) that compares odds of retention across predictor groups [63, 64]. These calculations were estimated assuming low percentage of the outcome given exposure (e.g., 15% uptake among Black PWID) to estimate the optimal sample size to conduct these analyses. To ensure that we can examine outcomes for Black, Latinx, women, and rural PWID in this analysis given the power calculation above, we have minimum sampling quotas of 20% of the total sample for Black PWID, 20% for Latinx PWID. 40% women, and 40% rural PWID, assuming overlap between these groups. This allows for exploratory subgroup analysis to examine differences within these subpopulations, but limits us to detecting larger odds ratio (2.5) and with approximately 78% power for Black and Latinx PWID.

## Discussion

The HOME study protocol details our approach to implementing a nationwide cohort of PWID, particularly those who have never previously engaged with mail-based harm reduction services. While there has been some studies using online-based recruitment of people who use drugs, many have noted the challenges of doing this work without prior guidance and a lack of best practices [65]. For our protocol, we have considered the best practices of conducting online-based surveys from previous work of recruiting transgender populations and men who have sex with men (including those who use drugs) for HIV-focused studies [30, 66–69] as well as recruitment strategies used among the few online-based surveys of people who use drugs [42, 59, 70]. One of the distinct challenges related to online-based recruitment is ensuring the study quality given the anonymity of online recruitment [71]. However, these concerns can be partially addressed by incorporating a rigorous protocol for identifying potentially fraudulent entries, such as collecting the same information at different points to check for consistency of responses, tracking IP addresses for multiple submissions, detecting the speed of survey completion, using software that reduces the risk of bots, and other approaches for mitigating fraudulent participation that we detailed in this protocol [71]. Data quality also needs to be assessed by examining missingness of data and consistency of responses across the surveys. While these concerns can be addressed, conducting online-based surveys of PWID requires significant resources for online-marketing efforts and detailed protocols prior to the start of data collection. We anticipate that our study will provide lessons learned and future guidance on best practices for recruiting large, underserved populations of PWID in an online setting.

For our study, we anticipate several potential challenges. First, recruiting Black and Latinx PWID via online spaces to obtain meaningful representation for subgroup analysis could be difficult. Given that no publication to our knowledge has provided guidance or best practices for online recruitment of Black and Latinx PWID, we will continually work closely with our community partners and CAB to identify online settings and marketing approaches to reach this population. Additionally, to address potential underrepresentation for Latinx PWID who do not speak English, a Spanish-speaking, culturally competent member of our research team is essential to identifying online spaces for recruiting Latinx PWID using our Spanish-translated ads. A second concern involves receiving overwhelming numbers of fraudulent eligibility surveys, such as bots completing hundreds of eligibility surveys at one time. Most importantly, this places significant burden on staff to identify fraudulent surveys on the back end using the tools and protocol that we have in place. While we plan to develop a machine learning approach for identifying bots and fraudulent entries, opening recruitment to large social media websites (e.g., Facebook) too soon has the potential to overwhelm staff and increase the wait time for eligible participants. Additionally, the sample recruited through this online-based methodology may underrepresent people who are unhoused or people with unreliable access to the internet, such as more rural regions of the country. We will collect information on housing status, geography, and internet access that will help us determine how our sample compares to other surveys of PWID in terms of recruitment bias. Finally, longitudinal cohort studies are well known to be challenging for obtaining high response rates at follow-up (i.e., retention), especially among PWID who may be transitory or involved with the criminal legal system. While we have multiple ways to contact the same person and even obtain contact information of a friend of the participant, we may still face more challenges with tracking down an online-recruited individual over time. Nonetheless, we plan to compare our response rates with other surveys in the field to assess whether our ability to do follow-up is substantially different from more traditional approaches.

Even with these challenges, there has been research signaling the potential for recruiting large national samples of people who use drugs and some successes with proper planning and resources [22]. At least one study has shown that among undergraduate students surveyed about drug use, web-based response rates were higher than mail-based response rates and the reported prevalence of drug use were not significantly different between web-based versus mail-based samples [72]. Moreover, this approach has the potential to reach people who are not engaged in community-based services and may have less access to services. While our study will provide data on the uptake and retention of PWID into mail-based harm reduction services, we will also generate needed information about the feasibility of using this methodology for national, large-scale recruitment of PWID for surveys in the future.

## Data Availability

Data sharing is not applicable to this article as no datasets were generated or analyzed during the current study.
